# Supplementary material for: Aptamer-Based Sensor for Rapid and Sensitive Detection of Ofloxacin in Meat Products
Source: Sensors (Basel). 2024 Mar 7;24(6):1740. doi: 10.3390/s24061740 (PMC10974795; doi:10.3390/s24061740)
Supplement: Supplementary file 1 [file sensors-24-01740-s001.zip › Highlights.pdf]

Highlights:

1. Aptamer-based sensor was developed for rapid on-site detection of ofloxacin.

2. Limit of detection of sensor was 0.61 nM of ofloxacin.

3. A fluorescent sensor designed for ofloxacin;

4. Direct detection of ofloxacin without complex pre-treatment.
