# Supplementary material for: Aptamer-Based Sensor for Rapid and Sensitive Detection of Ofloxacin in Meat Products
Source: Sensors (Basel). 2024 Mar 7;24(6):1740. doi: 10.3390/s24061740 (PMC10974795; doi:10.3390/s24061740)
Supplement: Supplementary file 1 [file sensors-24-01740-s001.zip › support information.pdf]

Aptamer-Based Sensor for Rapid and Sensitive Detection of Ofloxacin in Meat Products

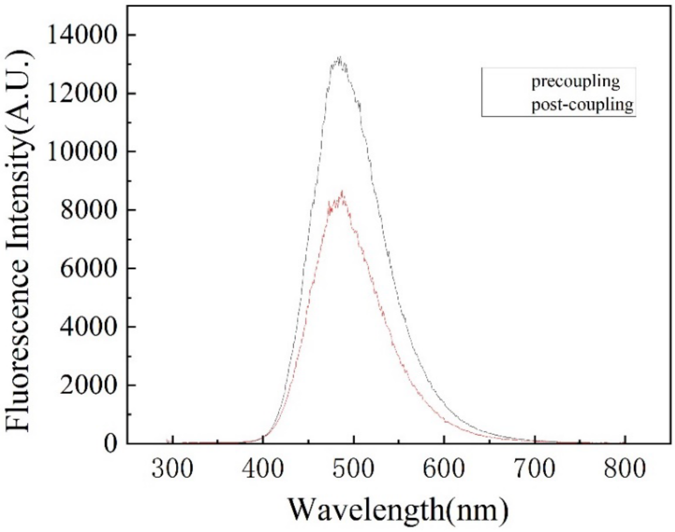

1 OFL coupling rates

Figure S1. OFL coupling rates.

2 Agarose gel electropherogram

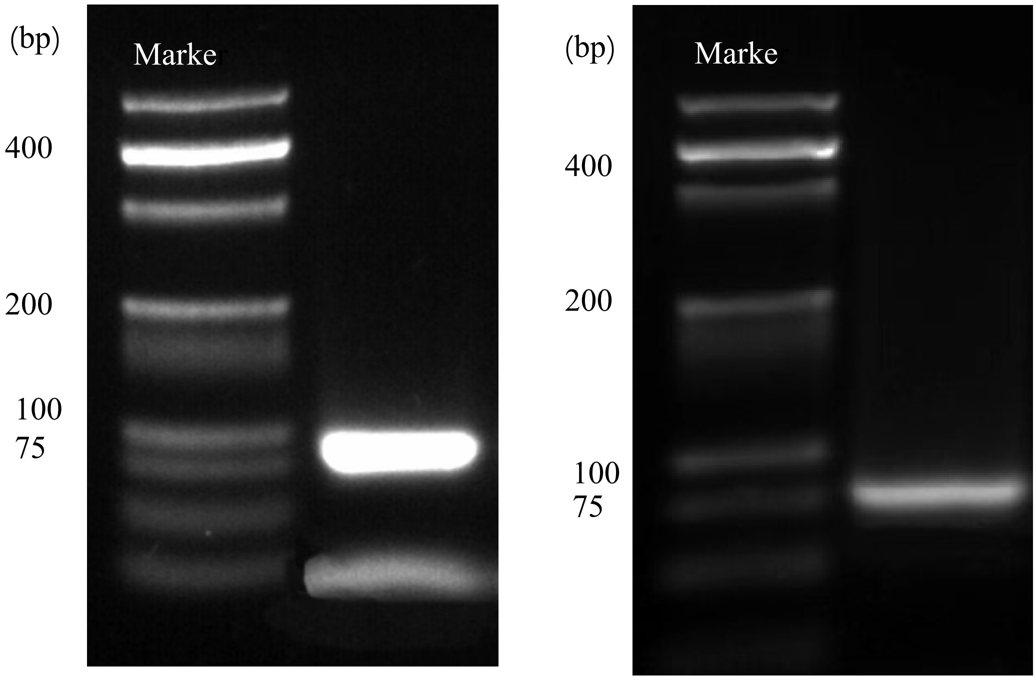

Figure S2. 3% agarose gel electrophoresis plots (A) Second round agarose gel electrophoresis plot; (B) Twelfth round agarose gel electrophoresis plot.

3 Meat High Performance Liquid Chromatography

3.1 HPLC conditions

The separation was performed on a themo scientific high performance liquid chromatograph (HPLC) with an Osaka Soda Capcell Pak C18 MG S-5 column (250 mm×4.6 mm, 5 μm) and methanol as the mobile phase at a detection wavelength of 280 nm at a flow rate of 0.3 mL/min and a temperature of 35 °C.The flow rate was 0.3 mL/min and the injection volume was 20 μL(Cheng et al., 2009; Wang and Gao et al., 2015; Zhang et al., 2016; Huang et al., 2021; Wang et al., 2021).

3.2 HPLC results

As can be seen from Figure S1, under the chromatographic conditions, there was no obvious interference peak at the retention time of OFL, and the peak shape was good. In the actual sample testing, no peak appeared at the retention time of OFL, indicating that the sample did not contain OFL.

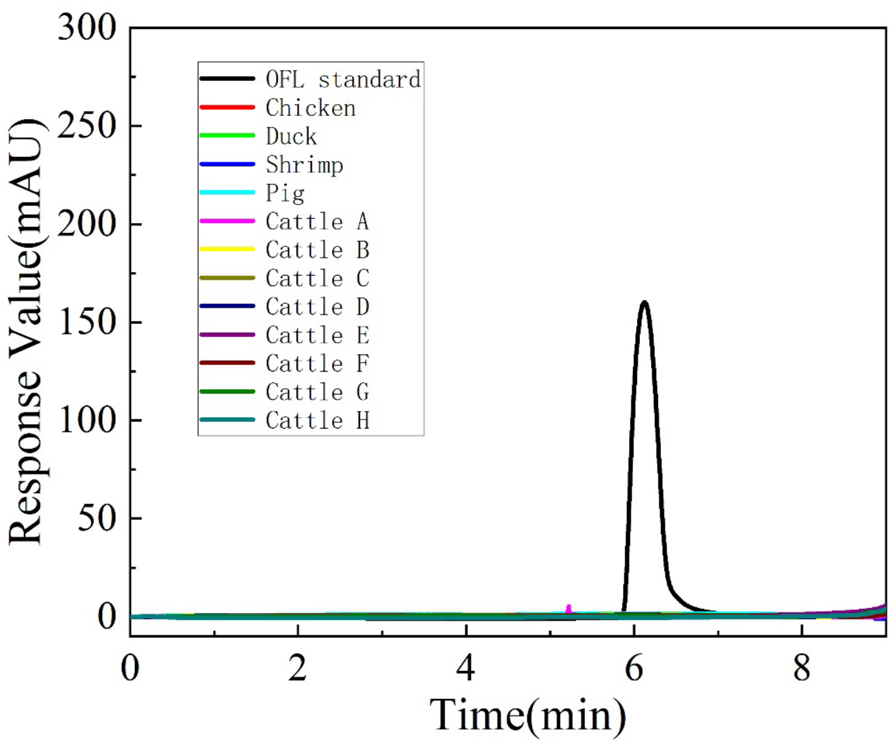

Figure S3. Results of high performance liquid chromatography.

References

Cheng, G. and H. Wu, et al. (2009). "Automated on-line microdialysis sampling coupled with high-performance liquid chromatography for simultaneous determination of malondialdehyde and OFL in whole blood." Talanta **79** (4): 1071-1075.

Huang, R. and Z. Guo, et al. (2021). "Assessment of veterinary antibiotics from animal manure-amended soil to growing alfalfa, alfalfa silage, and milk." Ecotoxicol Environ Saf **224**: 112699.

Wang, H. and M. Gao, et al. (2015). "Integration of phase separation with ultrasound-assisted salt-induced liquid–liquid microextraction for analyzing the fluoroquinones in human body fluids by liquid chromatography." Journal of Chromatography B **985**: 62-70.

Wang, R. and S. Li, et al. (2021). "Selective extraction and enhanced-sensitivity detection of fluoroquinolones in swine body fluids by liquid chromatography-high resolution mass spectrometry: Application in long-term monitoring in livestock." Food Chem **341** (Pt 2): 128269.

Zhang, Z. and X. Li, et al. (2016). "Multiresidue analysis of sulfonamides, quinolones, and tetracyclines in animal tissues by ultra-high performance liquid chromatography–tandem mass spectrometry." Food Chemistry **204**: 252-262.
